# Rapid Cycle Deliberate Practice Simulation for a Maternal Cardiac Arrest With Obstetrics and Gynecology Residents

**DOI:** 10.15766/mep_2374-8265.11513

**Published:** 2025-04-08

**Authors:** Timothy Friedmann, Ceyda Oner, Jared M. Kutzin

**Affiliations:** 1 Assistant Professor, Department of Emergency Medicine, Icahn School of Medicine at Mount Sinai; 2 Associate Professor, Department of Obstetrics, Gynecology, and Reproductive Sciences, Icahn School of Medicine at Mount Sinai; 3 Professor, Departments of Emergency Medicine and Medical Education, Icahn School of Medicine at Mount Sinai

**Keywords:** Cardiac Arrest, Obstetrics, Rapid Cycle Deliberate Practice, OB/GYN, OB/GYN, Critical Care Medicine, Simulation

## Abstract

**Introduction:**

Maternal cardiac arrest is rare; therefore, simulation serves as an opportunity to better prepare obstetrics and gynecology residents for these emergencies.

**Methods:**

We conducted a 2-hour educational activity for 16 obstetrics and gynecology residents at an academic medical center, utilizing rapid cycle deliberate practice to teach them the recognition and management of a maternal cardiac arrest. The case centered on a patient admitted to the labor and delivery floor who was in her third trimester. She developed chest pain and had a subsequent cardiac arrest. The case used up to seven rounds of rapid cycles with debriefing after each. Learners were expected to recognize the cardiac arrest, initiate initial management, consider the differential diagnosis, and prepare for a resuscitative hysterotomy.

**Results:**

After the simulation case, learners’ average comfort level for managing cardiac arrest improved from 1.8 to 3.6 (on a 5-point Likert scale: 1 = *extremely uncomfortable,* 5 = *extremely comfortable*). Comfort performing supportive airway management went from 1.9 to 4.1. Residents found the knowledge gained during the session useful to future practice, and the average course rating was 5.8 on a 6-point scale (with 6 = *excellent*).

**Discussion:**

Rapid cycle deliberate practice simulation for a maternal cardiac arrest improves obstetrics and gynecology residents’ comfort and readiness for obstetrics emergencies.

## Educational Objectives

By the end of this activity, learners will be able to:

1. Manage the initial resuscitation of a maternal cardiac arrest.
2. Apply basic and advanced cardiac life support skills to a pregnant patient.
3. Increase comfort in managing maternal cardiac arrest.
4. Review the differential diagnosis of maternal cardiac arrest.

## Introduction

In the United States, approximately one in 12,000 hospitalizations for delivery is complicated by maternal cardiac arrest.^1^ Obstetrics and gynecology (OB/GYN) residents must be prepared to recognize and initiate the management of a laboring patient who suffers from cardiac arrest. Because of the rarity of maternal cardiac arrest, simulation serves as an opportunity to better prepare OB/GYN residents for these emergencies. Prior work has shown that simulation of maternal cardiac arrest can improve resident knowledge and comfort levels.^2^

Rapid cycle deliberate practice (RCDP) is a medical simulation technique that utilizes cyclical, real-time debriefing. It allows learners to develop and iteratively improve skills based on debriefing points built into the simulation. Hunt and colleagues first described RCDP as a technique that is “focused on rapid acquisition of procedural and teamwork skills” and helps develop first-5-minutes skills for pediatric residents managing cardiac arrest.^3^ RCDP has also been shown to improve nursing education satisfaction and response to cardiac arrest events.^4^

Little has been published on RCDP in OB/GYN. One study examined RCDP versus traditional simulation for forceps deliveries done by residents. It found that both were effective in improving skills scores for forceps deliveries, although no difference was found between the RCDP and traditional simulation groups.^5^ Salvetti, Gardner, Minehart, and Bertagni studied labor and delivery clinicians managing critical events utilizing RCDP in a virtual environment.^6^ A literature search of *MedEdPORTAL* for *rapid cycle deliberate practice* returned seven publications utilizing RCDP.^7–13^ These primarily focused on pediatrics or emergency providers, with one centering on the operating room setting^11^ and another on outpatient cystoscopy for OB/GYN trainees.^13^ No cases specific to maternal cardiac arrest or obstetrics utilizing RCDP techniques were found in *MedEdPORTAL.* Our novel curriculum addresses this gap and contributes to the growing body of simulation work using RCDP.

Because RCDP had been successfully used in early resuscitation care for cardiac arrest events yet had not been applied to maternal cardiac arrest, we developed an RCDP simulation case to teach OB/GYN residents the initial resuscitation of a maternal arrest.

This simulation learning activity was developed as part of the preexisting OB/GYN resident simulation education curriculum in 2022, which consisted mainly of didactic lecture content and in situ simulations. Simulation and team training can strengthen institution readiness for maternal cardiac arrest. To prepare our residents for this acute emergency, we added cardiac arrest simulation education into our curriculum and implemented at least an annual event for learners. Obstetrical residents participate in traditional basic life support (BLS) and advanced cardiac life support (ACLS) training,^14,15^ which utilizes a component of simulation. Residents also utilize hemi-pelvic simulators to review obstetrics emergency procedures for shoulder dystocia and postpartum hemorrhage. Because residents are already training in BLS and ACLS and participating in team training scenarios about other obstetric emergencies, we felt that they would have the requisite knowledge to participate in an RCDP training regimen focused on maternal cardiac arrest. The use of RCDP is well suited to this population and this condition as the participants already have foundational knowledge but require the repeated practice of all the steps of managing a maternal cardiac arrest scenario.

## Methods

### Equipment/Environment

Simulation was conducted in the Simulation Teaching and Research (STAR) Center at the Icahn School of Medicine at Mount Sinai Hospital in New York, NY. We used Laerdal's SimMom, which was capable of delivering a baby and having chest compressions and defibrillation performed for the case. Given that not all high-fidelity obstetric features were necessary, this case could be run with any manikin capable of ACLS management with low-fidelity gravid abdomen modifications. Other equipment required was typical of ACLS cases: code cart with simulated medications, monitor equipment, defibrillator, oxygen, and basic airway management equipment, backboard, step stool, and a typical simulation room with a stretcher. While we did not allow the team to perform a resuscitative hysterotomy, we had povidone-iodine and a scalpel available in case they requested equipment to prepare for the procedure.

### Personnel

This simulation was designed to teach medical knowledge and improve comfort regarding the initial resuscitation of maternal cardiac arrest to several resident learners; therefore, we limited the additional staff in the simulation. Our target audience was OB/GYN residents at all levels of training. Some roles that might typically be held by nursing or other staff (e.g., recorder or compressor) were taken by the resident participants. This allowed both for a better understanding of roles in a code and for more learners to benefit from the simulation at once. We found that four to six participants at a time was useful.

This case required two additional personnel beyond the participants. One person (with a basic understanding of and ability to operate SimMom) managed the manikin, including vital signs, rhythm changes, and response to defibrillation attempts. The second was a standardized participant who served as the nurse. The standardized nurse participant assisted with medications (other than epinephrine, which was an explicit teaching point in this simulation), activated additional resources, and offered prompts to ensure forward progress during the simulation.

Our debriefing sessions were developed by faculty from multiple departments to facilitate an interdisciplinary approach. We had representation from the departments of obstetrics, gynecology, and reproductive sciences; emergency medicine; and anesthesiology. The session could be facilitated by one faculty member familiar with the content and RCDP.

### Implementation

Our RCDP case of maternal arrest was designed to teach the recognition and initial management of a maternal cardiac arrest. We aimed to teach OB/GYN residents the first 5 minutes of a resuscitation because we anticipated they would not have additional resources at the bedside in the initial resuscitation efforts.

The case was created by a multidisciplinary team including the senior director of simulation at emergency medicine, an emergency medicine medical education and simulation fellow, OB/GYN and anesthesia simulation faculty, and a safety nurse.

The session was scheduled to be conducted over 2 hours, during the usual protected teaching time slot for the residents. The case took place in the STAR Center in a standard patient room. The labor room was set up for the simulation by the three multidisciplinary facilitators and one simulation technician an hour in advance of the start of the session. We used the equipment described above.

Prior to the start of the activity, participants were assembled in the conference room, and a 10-minute prebriefing was conducted. During the prebriefing, participants were welcomed, and attendees were asked to individually introduce themselves by giving their name, where they were from, and a fun fact about themselves. Reassurance was provided to encourage a sense of psychological safety and emphasize the confidentiality of the learning activity. Participants were oriented to the manikin, room setup, and location of equipment.

Briefly, the simulation case (Appendix A) featured an expecting mother admitted to the labor and delivery floor who developed chest pain and shortness of breath. The nurse asked a resident to assess the patient; upon participant evaluation, the patient was in cardiac arrest. This multilearner RCDP case began with one resident in the room who was expected to recognize cardiac arrest and start compressions. They then called for help from the additional participants, who were waiting outside the room. Certain actions were expected prior to each round of debriefing (described in Appendix B). After each round of debriefing, the case was restarted with a new primary participant. They evaluated the patient, initiated cardiac arrest care, activated additional resources, and progressed through the case further than the previous participant had. Once they reached the designated point for debriefing, the case was again paused and debriefed as below. This continued until each participant had been the primary participant, and then the case concluded. Depending on the number of participants, an additional round could be added that focused on leadership and communication; once established, the code leader was blindfolded. This allowed a focused discussion on the importance of closed-loop and precise communication.

### Debriefing

This simulation utilized an RCDP debriefing strategy. We provided an overview of each round of critical items and the topics for debriefing once the simulation had been paused (Appendix B).

Round 1 consisted of the initial evaluation and recognition of cardiac arrest. The learner was expected to go through the basic evaluation of an unresponsive patient, check for a central pulse, initiate compressions, and call for additional help. The debrief typically focused on assessment of the patient.

The Round 2 debrief was broken into two main parts: assignment of roles and optimization of compressions. First, we discussed the roles required in cardiac arrest care. Depending on the exact number of participants, we could modify this to ensure that the numbers of participants and roles were equal, but we discussed the general roles in a cardiac arrest, as well as which were most important for the residents to do. We discussed the importance of leadership in cardiac arrest. The second focus of this debrief surrounded ensuring compression quality. This included preparing the bed by lowering the side rails, the head of the bed, and the bed from the wall. We also ensured that the leader would call for a step stool and backboard and that someone was assigned to uterine displacement.

Round 3 reviewed defibrillator management. We discussed the process of turning on the device, connecting the pads, pad placement, rhythm checks, and charging/shocking. We also debriefed compression and ventilation quality; each participant demonstrated high-quality compressions and appropriate bag valve mask ventilation techniques.

Round 4 focused on medication. We discussed medications used in cardiac arrest and the timing of administration. We also specifically reviewed epinephrine. We discussed concentration, dosing, and route of epinephrine. Finally, we reviewed the use of the Bristojet epinephrine injector system.

Rounds 5 and 6 focused specifically on maternal cardiac arrest, whereas the first several rounds were consistent with prior RCDP work for cardiac arrest. Round 5 debriefed on the decision to perform a resuscitative hysterotomy and the steps of the procedure. We specifically discussed timing in this round. Round 6 focused on the differential diagnosis for maternal cardiac arrest; this debrief allowed for a broad discussion on management of particular etiologies.

Finally, if time and number of participants allowed, Round 7 debriefed communication and leadership skills after the code leader had been blindfolded. This debrief also served as a global debrief for the entire exercise. We allowed for self-reflection, lingering questions, and discussion on any remaining medical knowledge or communication topics.

### Evaluation

This project evaluated the reactions to a novel simulation approach in our institution's simulation center housed in the emergency medicine department. The center had institutional review board (IRB) approval for evaluation of simulation exercises performed there, and this project fell under that IRB. Our standard simulation survey (Appendix C, p. 3) evaluated reactions to the simulation, including perceived knowledge gained, realism, and utility for practice.

Sixteen OB/GYN residents participated in the simulation. The participant group included first- through fourth-year residents. Out of the 16 participants, 12 had never seen a maternal cardiac arrest, and four had seen between one and five maternal arrests. Only one had previously participated in a maternal cardiac arrest simulation.

Our critical action checklist (Appendix B) was created by modifying the protocol from Kutzin and Janicke such that it was specific to maternal cardiac arrest.^3^

We also used case-specific pre- and postsimulation surveys (Appendix C), which were offered to all participants to evaluate effectiveness of the simulation. The presimulation survey was completed immediately prior to participation, and the postsimulation survey was administered immediately after participating in the session. Created by one of the authors specifically for this project, the survey tool assessed comfort levels on various topics on a 5-point Likert scale (1 = *extremely uncomfortable,* 5 = *extremely comfortable*). Due to discrepancies in the unique identifiers populated by participants, pre- and postsimulation survey data were not able to be linked by individual response. Therefore, we used Mann-Whitney *U* tests (using a freely available online calculator) to compare pre- and postsimulation comfort levels reported by participants.

## Results

Sixteen participants completed the presimulation survey, and 14 completed the postsimulation survey. The average comfort level reported for managing cardiac arrest was 1.8 in the presimulation survey and 3.6 in the postsimulation survey, a statistically significant difference (*U* = 19.5, *p* < .001). The comfort level with performing supportive airway management went from 1.9 to 4.1 (*U* = 18.5, *p* < .001). The Table shows the results for each comfort-level question; each shows a statistically significant improvement in comfort level after the simulation.

**Table.**
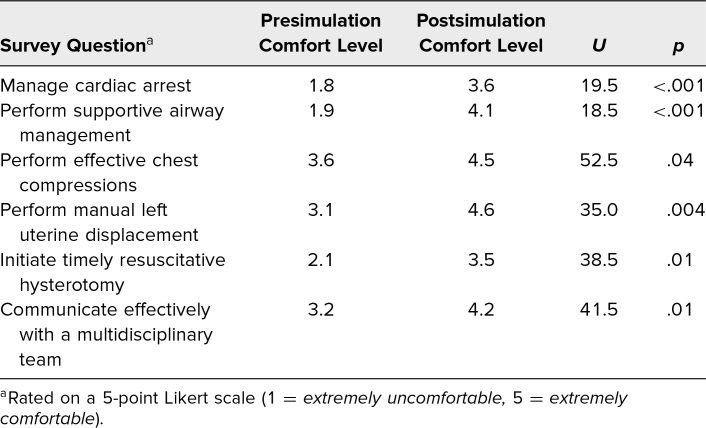
Comfort-Level Results

In each round of the simulation, facilitators assessed completion of critical actions and reviewed them in debriefs. Learners were able to ask questions, and facilitators addressed any knowledge gaps to ensure that all educational objectives were met. We discussed the recognition of cardiac arrest and focused on the initial management until additional resources could arrive. OB/GYN residents, the specific population here, are not expected to manage the entire resuscitation of patients suffering cardiac arrest, but it is imperative that they be able to initiate care for these patients. We discussed obstetric specific management including uterine displacement and resuscitative hysterotomy. Finally, we reviewed the differential diagnosis of an expecting mother in cardiac arrest.

Learners were asked to provide anonymous feedback about their perceptions of the simulation at the end of the session. Twelve participants completed the survey. The average rating on a 4-point scale (1 = *strongly disagree,* 4 = *strongly agree*) for “The knowledge I gained from the session will be helpful to me in my practice” was 3.7. The overall course rating on a 6-point scale (with 6 = *excellent*) averaged 5.8. Learners were asked to provide narrative comments on their biggest takeaways from the session. The three most common responses related to role assignment in cardiac arrest, initiation of compressions, and cardiac arrest care algorithms.

## Discussion

This RCDP simulation case offers a novel approach to an important emergent clinical scenario: maternal cardiac arrest. The case provides an effective educational opportunity for OB/GYN residents to learn the recognition and initial management of cardiac arrest. To our knowledge, this is the first application of RCDP to teach cardiac arrest care to OB/GYN residents.

Our simulation case worked well for our population and allowed for flexibility in teaching. The debriefing protocol and stop points can be modified depending on the number of learners in the case. The simulation allowed a group to learn together simultaneously without the resource burden of running a case for individuals. We feel that RCDP is an effective and efficient approach for teaching the initial management of cardiac arrest to multiple populations, including OB/GYN residents. Prior literature has shown the utility of RCDP in other settings, and our project demonstrates that learners feel this instructional method is both useful for future practice and enjoyable. Participants reported statistically significant increases in comfort levels for managing cardiac arrest, providing supportive airway management, performing effective chest compressions, performing manual left uterine displacement, initiating timely resuscitative hysterotomy, and communicating effectively with a multidisciplinary team. RCDP allows for these improvements using iterative, deliberate practice with a relatively large group of learners, thus avoiding overburdening educational resources. Additionally, while all learners were active throughout our session, prior simulation literature supports similar learning by observers and participants, potentially allowing for expansion of this session to include more learners or health care providers in different professions.^16^

Our project has several limitations. First, this case was offered to a small number of trainees. Despite statistical significance in our analysis, our small sample size and single center may hinder power and generalizability. Additionally, we had issues linking our pre- and postsimulation data, which limits utility; we had initially planned to use a Wilcoxon signed rank test rather than Mann-Whitney *U* but were unable to accurately link pre- and postsurveys by unique identifier code. This was a self-reported pre/post survey project that evaluated Kirkpatrick's level 1 outcomes only^17^; future work is required to assess more patient-centric outcomes. While we had multidisciplinary representation in terms of faculty physicians, this project did not include nursing staff, who are integral in the recognition and care of cardiac arrest patients. Finally, our project design did not include retention measurements; future studies should consider interval follow-up surveys to assess comfort levels after the activity.

Here, we have provided a detailed description of the design and implementation of multidisciplinary simulation training in maternal intrapartum cardiac arrest for residents. This session began as a yearly training within the simulation curriculum for the OB/GYN residency program. After its success with residents, we also ran it as a core learning activity for our physicians and labor nursing staff at our labor and delivery unit. Because of the natural decomposition in memory over time, regularly scheduled training in this infrequent but critical event is crucial to ensure preparedness. Because of this, we added the activity as part of our multidisciplinary obstetrics labor and delivery simulations in May 2023.

Anecdotally, we have received feedback from senior trainees indicating that they wished the training would occur closer to graduation so that they felt better prepared for this rare obstetrics emergency as they transitioned to being attendings.

Future work in this area could include nursing staffing or in situ cases to better simulate the staffing and environment of such clinical scenarios. RCDP has the potential to be useful in other populations and settings and should be considered in similar populations that must recognize and initiate care despite not being expected to lead the entire resuscitation.

## Appendices


Simulation Case.docxRCDP Debrief Guide.docxSurveys.docx
*All appendices are peer reviewed as integral parts of the Original Publication.*
