# Supplementary material for: Rapid Cycle Deliberate Practice Simulation for a Maternal Cardiac Arrest With Obstetrics and Gynecology Residents
Source: MedEdPORTAL. 2025 Apr 8;21:11513. doi: 10.15766/mep_2374-8265.11513 (PMC11975762; doi:10.15766/mep_2374-8265.11513)
Supplement: Supplementary file 1 — Simulation Case.docxRCDP Debrief Guide.docxSurveys.docx [file mep_2374-8265.11513-s001.zip › A. Simulation Case.docx]

| **Appendix A: Simulation Case**    **SIMULATION CASE TITLE: Maternal Cardiac Arrest**    **AUTHORS: Kutzin, J., Friedmann, T., and Oner, C.**    **LEARNER AUDIENCE: Obstetrics and Gynecology Residents**  **Activity Time: 2 hours** | |
| --- | --- |
| **PATIENT NAME: Ms. Jones**    **PATIENT AGE: 32**    **CHIEF COMPLAINT: Chest pain and shortness of breath**    **PHYSICAL SETTING: Labor and delivery room** | |
|  | |
| **Brief Narrative Description of Case** | The resident (learner) is summoned by nursing staff to see a 32 year old female admitted to the labor floor who was complaining of chest pain and shortness of breath. She is found to be in cardiac arrest. Learners should recognize and initiate treatment of cardiac arrest including activation of additional resources, ACLS care, and obstetric specific treatments. |
| **Primary Learning Objectives** | By the end of this activity, learners will be able to:  1. Initiate the management of a maternal cardiac arrest  2. Apply basic and advanced cardiac life support skills to a pregnant patient  3. Feel more comfortable in managing maternal cardiac arrest  4. Describe the differential diagnosis of maternal cardiac arrest |
| **Critical Actions** | 1. Recognize patient is unresponsive and perform basic assessment 2. Call for additional help 3. Recognize cardiac arrest 4. Begin and optimize compressions 5. Assign roles 6. Activate additional resources 7. Perform ACLS care including respiratory support, defibrillation, and medication administration 8. Perform uterine displacement 9. Consider resuscitative hysterotomy 10. Consider the differential diagnosis of maternal cardiac arrest 11. Use effective teamwork and communication |
| **Learner Preparation or Prework** | No learner preparation or pre-work is required for this case |

| **INITIAL PRESENTATION** | | | |
| --- | --- | --- | --- |
| **Initial Vital Signs** | **HR 0, BP 0/0, RR 0, T 37** | | |
| **Overall Setting and Appearance** | Learners are brought into a simulated labor and delivery room by the nurse who asks them to assess the patient. The mannequin is lying on a stretcher, unresponsive. | | |
| **Standardized Participants (and Their Roles in the Room at Case Start)** | The embedded nurse states “Can you please come assess Ms. Jones? She was complaining of chest pain and shortness of breath.” and leads the learner into the room. The nurse will remain in the room to assist as directed.  When the initial learner calls for help, the additional learners are brought into the room to assist as directed. | | |
| **HPI** | The patient is unresponsive and unable to provide history.  If asked, the nurse will state “She is 32 years old, G2P1 at 39 weeks and 6 days admitted here for labor. She has an epidural and had a top-off dose a while before she complained about chest pain and shortness of breath.” | | |
| **Past Medical/Surgical History** | **Medications** | **Allergies** | **Family History** |
| None. G2P1 | Top off dose 1.5 hours prior to complaining of chest pain and shortness of breath. No other meds. | None. | None. |
| **Physical Examination** | | | |
| **General** | Unresponsive | | |
| **HEENT** | Normal appearing | | |
| **Neck** | No JVD | | |
| **Lungs** | Apneic | | |
| **Cardiovascular** | Pulseless | | |
| **Abdomen** | Soft | | |
| **Neurological** | Unresponsive | | |
| **Skin** | Pale | | |
| **GU** | Normal | | |
| **Psychiatric** | N/A | | |

| **INSTRUCTOR NOTES - CHANGES AND CASE BRANCH POINTS** | | |
| --- | --- | --- |
| **Intervention / Time Point** | **Change in Case** | **Additional Information** |
| Pre-Case Set-Up | Equipment:   - SimMom (or other manikin capable of ACLS simulation and a low fidelity gravid uterus) - Code cart (with simulated ACLS medications) - Basic airway management equipment - Oxygen - Defibrillator - Backboard - Step stool - Stretcher - Standard simulation room equipment and monitors |  |
| Initial learner assesses patient and calls for help | Additional learners enter once they hear calls for help | No changes. Patient remains in cardiac arrest. |
| Cardiac arrest management | Roles:   - Leader - Compressor - Airway (x 2 when possible) - IV/Meds – place IV above diaphragm - Defib/Monitor/Code cart - Uterine Displacement   See Appendix B. for specific critical actions, roles, and debriefing instructions for each round. Each round will vary in time depending on the participants’ actions, but Appendix B lists critical actions with approximate times for each round. |  |
| Team asks for ICU/Anesthesia, calls code blue, activates additional hospital resources, etc. | RN states “they are tied up in an emergency in the OR / ICU. They will send someone as soon as they can.” |  |
| Team prepares for resuscitative hysterotomy | Case ends and team is prompted to discuss the steps of the procedure. |  |

**Ideal Scenario Flow**

The initial learner enters the room and assesses the patient. They recognize that the patient is unresponsive and taps them, shouts, and feels for a central pulse. They recognize that the patient is in cardiac arrest and simultaneously begins compressions and calls for additional help. The rest of the learners arrive. The initial learner assigns roles including a leader. The team optimizes the patient and room for compressions and cardiac arrest care: they lower bed rails, lower the head of the bed, move the bed out to make room for airway management, and lower the bed height. They also bring a code cart, backboard, and a step stool. Someone is assigned to perform uterine displacement in addition to typical cardiac arrest roles. The team provides high-quality ACLS care with effective teamwork and communication. They activate additional resources to help manage the patient. Within five minutes, they consider and decide to perform an emergent resuscitative hysterotomy.

**Anticipated Management Mistakes**

*Provide a list of management errors or difficulties that are commonly encountered when using this simulation case.*

1. Recognition of cardiac arrest: We found that learners often ask for the nurse to place the patient on the monitor before recognizing unresponsiveness. We also noticed that many learners will palpate peripheral pulses rather than central ones (carotid or femoral).
2. Calling for help: Some of our learners did not shout for help or press the code button on the wall; we remind them in the first debrief the importance of bringing more people to the bedside by shouting loudly to get attention.
3. Optimizing compressions: Many learners needed reminders on how to best do compressions. Debrief two focuses on the actions and items necessary to perform effective compressions including bed positioning, backboards, etc. Learners were reminded during round 2 that chest compression hand placement should be at the lower sternum rather than mid-sternum in the pregnant patient.
4. Assignment of roles: Most of our learners assumed the role of leader, but occasionally, they continued doing compressions and didn’t assign a leader. In round 3, we talk about assigning roles, handing off tasks, and ensuring there is someone assigned to each critical role.
5. Defibrillator management: This mistake may vary by staff familiarity and model of defibrillator. We find that many learners in this population are not comfortable using the defibrillator that is throughout our institution; most commonly, learners had difficulty with connecting pads and defibrillator modes.
