# Supplementary material for: Rapid Cycle Deliberate Practice Simulation for a Maternal Cardiac Arrest With Obstetrics and Gynecology Residents
Source: MedEdPORTAL. 2025 Apr 8;21:11513. doi: 10.15766/mep_2374-8265.11513 (PMC11975762; doi:10.15766/mep_2374-8265.11513)
Supplement: Supplementary file 1 — Simulation Case.docxRCDP Debrief Guide.docxSurveys.docx [file mep_2374-8265.11513-s001.zip › B. RCDP Debrief Guide.docx]

**Appendix B. Debrief Guide**

**Background on Rapid Cycle Deliberate Practice:**

Traditional simulation has been used to train EM residents in many skills, including cardiac arrest care. Traditional simulation consists of a medical case that is presented to learners, simulated by learners in full, and is followed by a debriefing session to discuss various aspects of the case including medical decisions, communication, and teamwork. In contrast, rapid cycle deliberate practice (RCDP), first described by Hunt et al., utilizes a learning theory-based approach that utilizes directed and deliberate practice to build automatization and mastery of skills and emphasizes psychological safety (Hunt et al., 2014).

**How to facilitate RCDP:**

Rapid cycle deliberate practice simulations use a cyclical approach to a given case. The facilitator will start a case in a similar fashion to a traditional simulation: the learner(s) will participate in a pre-brief and then the case is introduced. The case will run until pre-specified stop points so that the components are broken into more digestible segments with feedback integrated into the case after each segment. After each segment, the case is reset and restarted from the beginning. The case runs beyond the first debriefing point and continues to the next; this is repeated until the case is completed. This can be done with a single learner, or multiple learners can participate at the same time.

**This case:**

Refer to the Case Template (Appendix A) for details on the clinical scenario presented to participants. A single participant is participant is brought into the room by a nurse (an embedded participant who leaves immediately) and is asked to assess a patient admitted to the labor and delivery floor who recently complained of chest pain and shortness of breath. The patient is unresponsive and is suffering from cardiac arrest. The participant should assess the patient, check for a pulse, activate additional resources (including bringing the additional participants into the room to help), and begin compressions. Once they call for help and the rest of the team joins, the case is paused. Round 1 debrief should focus on:

Round 1

Critical Actions:

- Tap and Shout
- Check a Pulse – carotid or femoral
- Call for help
- Start Compressions

Debriefing instructions:

Round 1 consists of the initial evaluation and recognition of cardiac arrest. The learner is expected to go through the basic evaluation of an unresponsive patient: this includes speaking (and subsequently shouting) to the patient, tapping and/or sternal rub, and checking for a pulse. This debrief reviews the importance of escalating stimuli in order to awaken a patient and assess for true unresponsiveness. Review the importance of checking a central pulse at either the carotid or femoral pulse because a profoundly hypotensive patient may not have a palpable peripheral pulse but does not require cardiopulmonary resuscitation. We found that learners may ask the nurse to connect the patient to the monitor or for vitals before checking a pulse. They often will check a radial pulse first. Remind them that in an unresponsive patient, a pulse check must take priority over attachment to the monitor.

Review ways to bring additional resources to the bedside. Upon discovering the unresponsive, pulseless patient, participants should shout loudly for help. They should avoid leaving the patient’s bedside unless absolutely necessary to get more help and should prioritize initiation of compressions.

After Round 1 debrief, the case is then reset. A new participant from the group is selected to begin the case. The same introduction is made by the nurse and the participant will begin the case from the start. They will continue the case beyond Round 1 stop point, allowing the remainder of the participants to join when they call for help. Participants will begin to work as a team to provide cardiac arrest care. The case should continue for ~1-2 minutes after entrance of the entire team before stopping for Round 2 debrief which focuses on:

Round 2

Critical Actions:

- Put side rail down
- Put head of bed down
- Start Compressions
- Assign Roles
  - Leader
  - Compressor
  - Airway (x 2 when possible)
  - IV/Meds – place IV above diaphragm
  - Defib/Monitor/Code cart
  - Uterine Displacement
  - C-section supplies / Scalpel and Betadine
- Call for more help – neonatology/pediatrics, anesthesia, code team – to get adult ICU assistance
- First few steps before IV/Meds & Defib
  - Move bed away from wall
  - Backboard under patient
  - Step stool

Debriefing instructions:

Round 2 debrief is broken into two main parts: assignment of roles and optimization of compressions. First, we discuss the roles required in cardiac arrest care: team leader, timer/recorder, compressions, airway, access/medications, defibrillator/monitor, runner, etc. In this case, we also discuss the importance of assigning someone for uterine displacement given the gravid uterus impeding venous return. Depending on the exact number of participants, we can modify this to ensure the number of participants and the roles are equal, but we discuss the general roles in a cardiac arrest and which are most important for the residents to do. We discuss the importance of leadership in cardiac arrest and emphasize closed-loop communication. We also discuss prior code experiences and when roles worked well versus led to challenges. Encouraging the initial participant at the start of each round to be the code leader allows for all participants to lead at least once.

The other focus of this debrief surrounds ensuring compression quality. This includes preparing the bed by lowering the side rails, lowering the head of the bed, and the bed from the wall. We also ensure the leader is calling for a step stool and backboard. We emphasize the importance of high-quality basic life support (BLS) care. Fetal monitoring equipment should be removed to allow for resuscitation.

They should also activate the code team (the debrief should include institution specific instructions for doing so such as wall-based code buttons, emergency operators, etc.). After the Round 2 debrief, the case should be reset with a different initial participant and should run from the beginning through a pulse check – the goal of Round 3 Debrief is to focus on defibrillator use and CPR feedback.

Round 3

Critical Actions:

- Allow to attempt to hook-up pads and turn on defibrillator
- Rhythm check and shock if indicated
- Review components of high-quality CPR
  - Allow each person to demonstrate high-quality compressions during debrief
  - Allow each person to demonstrate adequate bag-valve mask ventilations during debrief
- Look at defibrillator for CPR feedback

Debriefing instructions:

Round 3 debrief reviews defibrillator management (our institution uses Zoll). We discuss the process of turning on the device, connecting the pads, pad placement, and selecting the correct mode (defibrillation rather than pacing or monitor). We review rhythm checks and how to maximize the compression fraction; we discuss ensuring that hands are on the pulse prior to pulse check. We also review how to charge and administer shocks with the defibrillator, ensuring that safety of the team is a priority. Finally, we debrief compression and ventilation quality; each participant demonstrates high-quality compressions and appropriate bag valve mask ventilation techniques. We demonstrate the CPR feedback capabilities of our defibrillator. After Round 3 debrief, the case is reset with a new initial participant and runs through the second pulse check to allow a discussion on medications in Round 4 debrief.

Round 4

Critical Actions:

- Epinephrine administration
  - Bristojet set-up and use
  - Route
  - Dosage
  - Frequency

Debriefing instructions:

Round 4 debrief focuses on medication. We discuss common medications used in cardiac arrest and the timing of administration within the ACLS algorithm. We specifically review epinephrine because it is the only necessary medication for this patient presentation. We review the concentration, dosing, and route of administration. We also discuss the logistics of who gives medications in codes, how to ensure the right medication/dosage/route is used, and how to communicate medication administration to the team leader. Finally, we review the use of the Bristojet epinephrine injector system and allow all participants to practice setting up and administering epinephrine using it.

The case is reset and a new initial participant starts the case from the beginning again; the case proceeds until five minutes into the cardiac arrest.

Round 5

Critical Actions:

- Prepare for resuscitative hysterotomy
  - Scalpel and betadine
  - Decision making
  - Procedure steps

Debriefing instructions:

Round 5 debrief marks the beginning of the specific focus on the obstetric population. It reviews the decision to perform a resuscitative hysterotomy, when to do it, and the steps of the procedure. Because the focus of this session is on the initial resuscitation of cardiac arrest patients and is aimed to improve comfort and familiarity of OBGYN residents of ACLS for their patient population, we felt that the decision to perform the procedure was the most important aspect and opted to exclude the procedure itself. We felt it was important to discuss the timing and how to facilitate the logistics of the procedure but avoided additional cognitive load with a procedure during the case. While a resuscitative hysterotomy was not performed, this session could be easily modified to include a partial-task trainer during the Round 5 debrief. After Round 5 debrief, the case is again reset with a new initial participant and restarted. The case runs from the beginning through 3 pulse checks.

Round 6

Critical Actions:

- Review and address any identified causes of maternal arrest:
  - A Anesthetic complications
  - B Bleeding
  - C Cardiovascular
  - D Drugs
  - E Embolic
  - F Fever
  - G General non-obstetric causes of cardiac arrest (H’s and T’s)
  - H Hypertension

Debriefing instructions:

Round 6 focuses on the differential diagnosis for maternal cardiac arrest and the importance of considering and addressing any potential causes during the case. During this debrief, we review a mnemonic that covers the broad scope of potential etiologies. During each, we review specific causes and their potential management. We put this differential into the context of the patient presentation and in this case, the etiology is not clearly one specific cause, but the debrief discusses which are more likely than others. After this discussion, the case is reset a final time. Depending on the number of participants, the cycle of initial participants may be restarted. The case runs through at least 3 pulse checks and allows the team to synthesize all of the prior rounds. As soon as a leader is identified, the simulation operations team presents a blindfold to the leader and asks them to put it on. The case runs while the leader is blindfolded.

Round 7

Critical Actions:

- Leadership
  - Blindfolding – how did that change things?
- Teamwork
- Communication

Debriefing instructions:

Round 7 debriefs communication and leadership skills after the code leader is blindfolded. We review communication among the team and the blindfold serves as a reminder to be as precise as possible while communicating, always ensuring closed loop communication. This debrief also serves as a global debrief for the entire exercise; we allow for self-reflection, lingering questions, and discussion on any remaining medical knowledge or communication topics. Participants are asked to describe their takeaways from the session before it concludes.

**References:**

1. Hunt EA, Duval-Arnould JM, Nelson-McMillan KL, et al. Pediatric resident resuscitation skills improve after "rapid cycle deliberate practice" training. Resuscitation. 2014;85(7):945-951.
