# Supplementary material for: Rapid Cycle Deliberate Practice Simulation for a Maternal Cardiac Arrest With Obstetrics and Gynecology Residents
Source: MedEdPORTAL. 2025 Apr 8;21:11513. doi: 10.15766/mep_2374-8265.11513 (PMC11975762; doi:10.15766/mep_2374-8265.11513)
Supplement: Supplementary file 1 — Simulation Case.docxRCDP Debrief Guide.docxSurveys.docx [file mep_2374-8265.11513-s001.zip › C. Surveys.docx]

**Pre-Intervention Survey**

1. Please enter the number of your home address followed by the second digit of your age (to create a unique identifier. i.e. If you live at 245 North Ave and are 32 years old, enter 2452).
2. Have you participated in the Maternal Cardiac Arrest simulation before?
   - Yes
   - No
   - Maybe
3. Please select your year in residency:
   - PGY-1
   - PGY-2
   - PGY-3
   - PGY-4
4. How many maternal cardiac arrests have you encountered?
   - 0
   - 1-5
   - 6-10
   - 11+
5. How comfortable do you feel with each of the following?
   - Manage cardiac arrest
   - Perform supportive airway management
   - Perform effective chest compressions
   - Perform manual left uterine displacement
   - Initiate timely perimortem cesarean section
   - Communicate effectively with a multidisciplinary team

Responses:

- - Extremely UNcomfortable
  - Somewhat UNcomfortable
  - Neither comfortable nor uncomfortable
  - Somewhat COMfortable
  - Extremely COMfortable

**Post-Intervention Survey**

1. Please enter the number of your home address followed by the second digit of your age (to create a unique identifier. i.e. If you live at 245 North Ave and are 32 years old, enter 2452).
2. How comfortable do you feel with each of the following?
   - Manage cardiac arrest
   - Perform supportive airway management
   - Perform effective chest compressions
   - Perform manual left uterine displacement
   - Initiate timely perimortem cesarean section
   - Communicate effectively with a multidisciplinary team

Responses:

- - Extremely UNcomfortable
  - Somewhat UNcomfortable
  - Neither comfortable nor uncomfortable
  - Somewhat COMfortable
  - Extremely COMfortable

**Standard Simulation Center Survey**

1. Submission Date
2. Learner Type
3. What department do you work in?
4. Department
5. What year are you?
6. Years in Practice (post residency)
7. Professional Role
8. Course Name
9. Instructor's Last Name
10. The instructor informed me of the goals and objectives of the session.
11. The instructor was able to answer my questions.
12. My simulation instructor was organized and prepared.
13. The orientation that I received to the environment and equipment was sufficient (I knew what the equipment could and could not do, how not to damage equipment, etc).
14. How realistic did you feel the simulation experience was?
15. The debriefing session enhanced my knowledge.
16. The knowledge I gained from the session will be helpful to me in my practice.
17. What I learned today will help to improve patient outcomes.
18. Overall this course was:
19. My biggest take home from this course was:
20. Please list any other topics/courses you would like to have offered at the STAR Center.
21. Any confidential information you would like to provide to the Center Director
